# Human-like scene interpretation by a guided counterstream processing

**DOI:** 10.1073/pnas.2211179120

**Published:** 2023-09-28

**Authors:** Shimon Ullman, Liav Assif, Alona Strugatski, Ben-Zion Vatashsky, Hila Levi, Aviv Netanyahu, Adam Yaari

**Affiliations:** ^a^Department of Computer Science, the Weizmann Institute of Science, Rehovot 76100, Israel; ^b^Computer Science and Artificial Intelligence Laboratory, Massachusetts Institute of Technology, Cambridge, MA 02139

**Keywords:** scene perception, scene understanding, top–down processing, combinatorial generalization, guided vision

## Abstract

In modeling vision, there has been a remarkable progress in recognizing a range of scene components, but the problem of analyzing full scenes, an ultimate goal of visual perception, is still largely open. To deal with complete scenes, recent work focused on the training of models for extracting the full graph-like structure of a scene. In contrast with scene graphs, humans’ scene perception focuses on selected structures in the scene, starting with a limited interpretation and evolving sequentially in a goal-directed manner [G. L. Malcolm, I. I. A. Groen, C. I. Baker, *Trends. Cogn. Sci.* **20**, 843–856 (2016)]. Guidance is crucial throughout scene interpretation since the extraction of full scene representation is often infeasible. Here, we present a model that performs human-like guided scene interpretation, using an iterative bottom–up, top–down processing, in a “counterstream” structure motivated by cortical circuitry. The process proceeds by the sequential application of top–down instructions that guide the interpretation process. The results show how scene structures of interest to the viewer are extracted by an automatically selected sequence of top–down instructions. The model shows two further benefits. One is an inherent capability to deal well with the problem of combinatorial generalization—generalizing broadly to unseen scene configurations, which is limited in current network models [B. Lake, M. Baroni, *35th International Conference on Machine Learning, ICML 2018* (2018)]. The second is the ability to combine visual with nonvisual information at each cycle of the interpretation process, which is a key aspect for modeling human perception as well as advancing AI vision systems.

## Significance

Understanding a visual scene is an unsolved and daunting task, since scenes can contain a large number of objects, their properties, and interrelations. Extracting the full scene structure is therefore infeasible, but often unnecessary, since it will be sufficient to extract a partial scene structure, which depends on the observer’s goal and interest. The presented model has a human-like ability to perform such a partial interpretation, focusing on scene structures of interest, evolving sequentially, in a goal-directed manner. The model uses a cortex-like combination of bottom–up and top–down networks, where the goal is achieved by automatically providing a sequence of top–down instructions that guide the process in an efficient manner, which generalizes broadly across different scene structures.

Scene understanding requires the extraction and representation of scene components, such as objects and their parts, people, and places, together with their properties and relations between them (1–4). Over the past several years, there has been a remarkable progress in using deep-network models for recognizing a range of scene components (5, 6) and pairwise object relations (7); however, the problem of analyzing full scenes, leading to scene understanding, is still largely open. To deal with complete scenes, recent work has focused on the training of models for extracting the full scene structure, producing a “scene graph,” which represents in a graph form all the scene components, properties, and relations (1, 4, 8). In contrast with extracting in parallel a full scene structure, humans’ scene perception is partial, focusing on selected structures in the scene, starting with a limited interpretation [the scene “gist” (9, 10)] and evolving sequentially over time, often in a goal-directed manner (2, 9–14).

Extracting a full scene structure of a complex natural scene is in general both infeasible (due to the full graph size) and often unnecessary (given the observer’s goal). The selective interpretation produced by human perception therefore offers an attractive alternative to current models, but it also raises new difficulties, in particular extracting selected scene structures of interest in a goal-guided manner, and applying a sequential process that depends on both the image content and the current goal. We propose a model that performs a human-like interpretation by combining bottom–up (BU) and top–down (TD) streams with bidirectional interactions, consistent with the two streams of the cortical “counterstream” structure (15–17). We describe below the model structure, its training procedure, and how it extracts scene structure in a goal-directed manner, using automatically generated sequences of TD instructions. We also make comparisons to the possible use of vision-language models for extracting scene structures of interest.

## Results

**Fig. 1.**
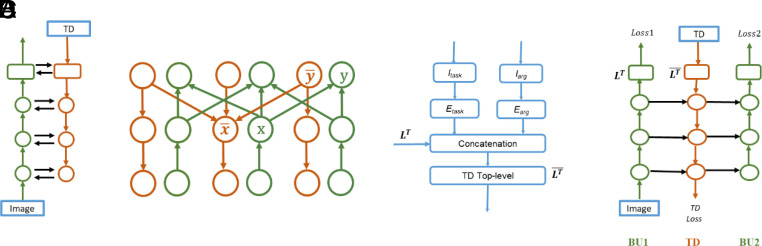
The BU-TD counterstream structure. (*A*) The two streams, input to the BU stream (green, pointing up) is the image; input to the TD stream (red, pointing down) is a TD instruction. Each circle is a complete layer, black arrows are cross-stream connections. (*B*) Interconnections within the streams. Each circle is a neuron in the network, x, y on the BU stream, $\bar{x}$   , $\bar{y}$   on the TD; cross-stream connections (omitted from figure) connect corresponding units in both directions (*SI Appendix*, *Cross-Stream Lateral Connections and Losses*). (*C*) Providing TD instructions. *I*_task_, *I*_arg_ are the task and argument instructions; *E*_task_, *E*_arg_, are their learned embedded vectors. $L^{T}$ : top-layer of the BU stream, $\bar{L^{T}}$ : input layer of the TD stream. (*D*) Training of the BU-TD network, using “unfolding in time”; BU2 is the same as BU1 at a later time, therefore BU1, BU2 share weights. Detailed structure in *SI Appendix*, Fig. S1). The final loss in training is at the top of BU2, two additional losses can be used at the top of BU1 and the bottom of TD (*SI Appendix*, *Cross-Stream Lateral Connections and Losses*).

We describe below the structure of the scene interpretation model, followed by its application to goal-directed scene analysis and its ability to perform combinatorial generalization. The model is comprised of BU and TD components that are identical in structure, but directed in opposite directions. The BU stream is modeled as a standard deep net (using, e.g., ResNet, ref. 18). A schematic representation of the BU-TD structure is shown in Fig. 1 *A* and *B*, a fuller description is given in *SI Appendix*, Fig. S1 and *Cross-Stream Lateral Connections and Losses*).

The model receives two inputs: an image to the BU stream, and a TD instruction to the TD stream (Fig. 1*A*). The TD instruction is provided to the model by two input vectors (Fig. 1*C*), one specifies a task to perform (denoted *I*_task_), the second (*I*_arg_) specifies an argument, (i.e., the object to apply the task to). *I*_task_ was specified to the model by a single “1” in the k’th entry for the k’th task in a set of learned tasks (e.g., “right-of,” “holding”). Similar to human vision (19), the argument *I*_arg_ can be specified in several ways: by an object’s location (e.g., a given image coordinate or region), similar to spatial attention, by an object identity (e.g., “Table”), similar to object-based attention, as well as by a relation, such as “right-of,” “occluded-by” another object (*SI Appendix*, Fig. S2). During training, *I*_task_, *I*_arg_ are transformed into new learned “embedded” representations *E*_task_*, E*_arg_. The learned representations are combined with the top-level image representation produced by the BU stream (*L^T^* in Fig. 1), and then propagate through all layers of the TD stream. Through the cross-stream connections, the next BU pass will take place in the context of the current TD activation; it is consequently controlled and guided by the TD instruction. Training the model was accomplished using standard methods for recurrent versions of deep networks (20, 21) using an “unfolded” version of the network (Fig. 1*D*).

Training was performed on all the individual tasks, which are later used for the extraction of scene structures. These include the recognition of all the different classes of scene components, their properties and pairwise relations. Training for the individual tasks is sufficient, the model can subsequently extract structures of interest without training with examples of specific structures. During training, we repeatedly sample a scene and a task, provide the TD instruction (*I*_task_, *I*_arg_) and the relevant ground truth label, and use the output to update the model.

Following training, a TD instruction can modify the BU stream to perform the selected task with high task selectivity (22), and sequences of learned TD instructions can be applied by the BU-TD model to perform complex visual tasks. For additional details on training the network and performance, see *Materials and Methods, Scenes Dataset*.

We describe next the extraction of scene structures of interest in a goal-directed manner, using automatically generated sequences of TD instructions. For general model testing, we also applied it to the “full structure” task, extracting all the scene components, their properties and relations as described in *SI Appendix*, *Extracting Full Scene Structure* and Fig. S3.

**Fig. 2.**
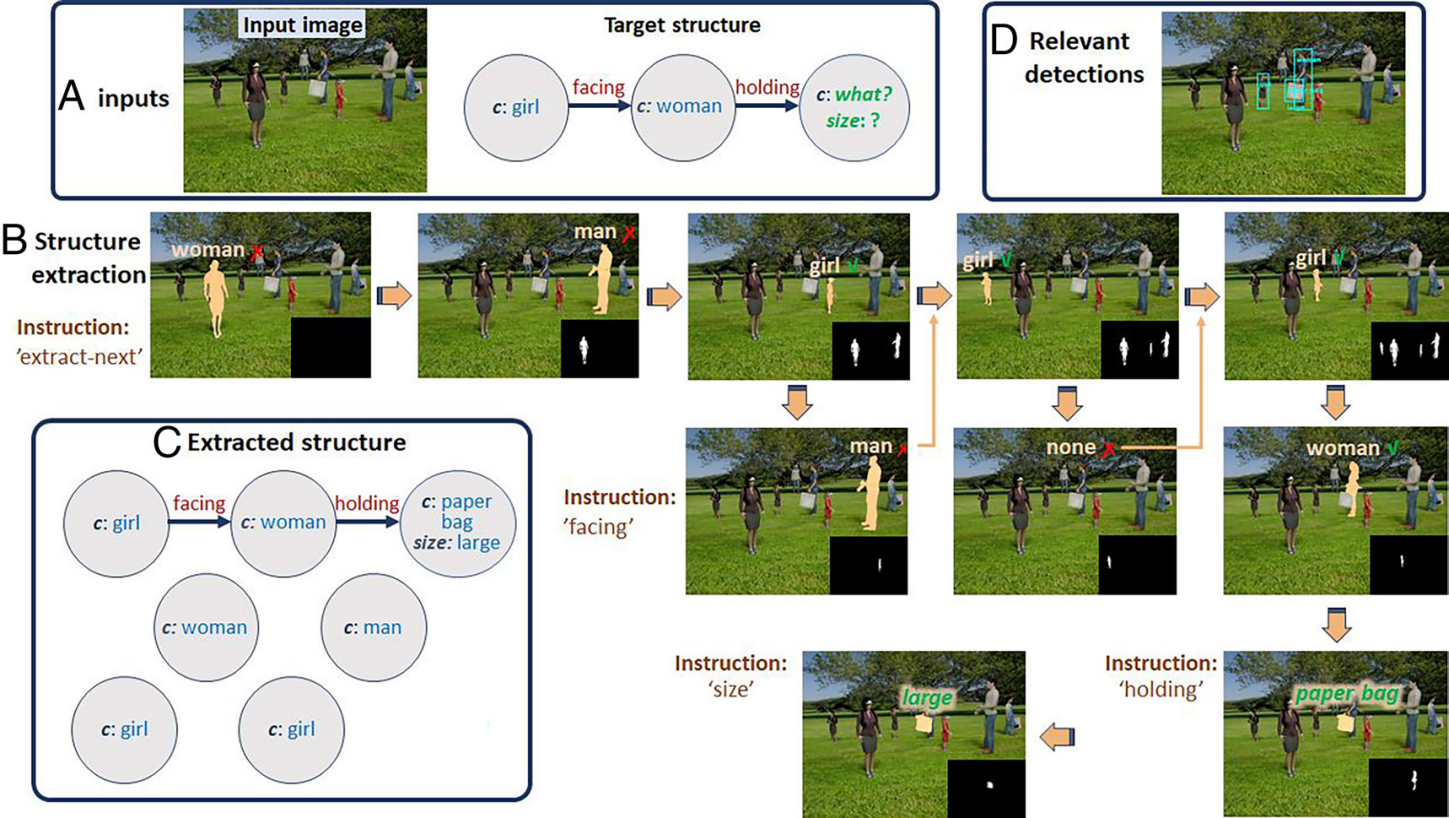
Goal-directed extraction of a scene structure. (*A*) Input scene and a target structure representing the current goal (a woman facing a girl and holding an object, identifying the held object and its size). (*B*) The extraction process guided by an automatically generated sequence of TD instructions, proceeding as indicated by the yellow arrows, left to right, top to bottom, showing the TD instructions, segmentation map supplying the arguments (*Insets*), and result of each step (highlighted). Results of the final two steps (paper bag, large; in green) extract the missing part of the target structure. (*C*) Extracted output structure. In terms of components, properties and relations, the extracted structure is a fraction of the full scene structure. (*D*) Grounding: image locations of the relevant extracted components (shown by bounding boxes). Green v symbols indicate that the expected object class or property were detected, red x symbols indicate that the expected class or property were not detected.

The scene images used in this work were computer-generated, with multiple persons and objects; this allowed us to use complex scenes with known ground truth for training and evaluation (*Materials and Methods, Scenes Dataset*). An example of applying a BU-TD sequence to a scene in a goal-directed manner is shown in Fig. 2. Rather than extracting a full structure, the current goal guiding the process is represented by a target structure of interest: Find in the image what the woman facing the girl is holding and its size (Fig. 2*A*), locating and identifying all the relevant components. Alternative modes of specifying the goal can be used, and the scene can also be approached without an explicit goal, relying instead on default priorities (*SI Appendix*, *Providing Goals* and Fig. S4*C*).

Fig. 2*B* shows the sequence of TD instructions used to extract the structure of interest, produced automatically by the model. The first TD task instruction (in Fig. 2*B*, top-left) is “extract-next” (extracting the next person/object in order of distance from the camera); argument is provided by an input-mask (bottom-right inset in each image), initially empty. The step’s output is the closest person to the camera, segmented, and classified (“woman,” highlighted). A series of “extract-next” and “facing” instructions eventually identify a girl and woman facing each other (last column), and the process continues to completion (holding a paper bag, large). The extracted scene structure (in Fig. 2*C*) is stored in the form of an array of components with their associated information (properties, relations, and segmentation maps); the structure extracted to reach the goal is only a fraction of the full scene structure. The output also provides a “grounding” of the extracted structure in the image, by associating each item with its segmentation map. The grounding can be subsequently used to extract additional properties and relations of selected components (*SI Appendix*, Fig. S4*B*), or to add nonvisual properties and relations retrieved from prior knowledge (2). Fig. 2*D* illustrates the grounding schematically by showing the image locations of the relevant target components.

The procedure used to produce the next TD instruction is a recursive process that handles one node at a time in a depth-first order (details in *Materials and Methods, Guided Structure Extraction and Selecting the Next TD Instruction*). The process is carried out by the combination of two subnetworks: an “expansion” and an “elaboration” network. The expansion network adds a novel scene component (person or object) to the extracted graph, based either on its relation with a reference (e.g., <facing, person-1> will add the person facing the reference person), or on its distance from the camera, using the <extract-next> instruction. The elaboration network is used for elaborating the already existing graph, by extracting additional properties and relations specified by the TD instruction. For details on the networks and their performance, see *Materials and Methods*, *Scene Dataset*, for details of the instruction selection algorithm see *SI Appendix*, *Extracting Full Scene Structure* and Fig. S3. Building upon the algorithm in ref. 23, the TD instruction selection process above can also deal with structures of interest that include the use of logical connectives (e.g., “a car or a bicycle”) and quantifiers (e.g. “all the girls”) (*SI Appendix*, Algorithm S2 and Fig. S4*A*).

The inclusion of quantifiers broadens significantly the scope of the interpretation and makes it more human-like compared with alternative approaches (24) (*SI Appendix*, *Comparisons with Alternative Models*).

### Combinatorial Generalization.

**Fig. 3.**
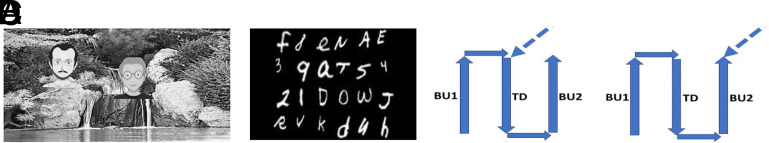
Combinatorial generalization. (*A* and *B*) Example inputs for two tasks. (*A*) Recovering the persons’ identities and their properties. (*B*) Identifying left- or right-of neighbor of handwritten characters. (*C* and *D*) Schematic backbones of alternative models: (*C*) BU-TD guided, (*D*) unguided (readout selection); arrows point to the location of providing the instruction [input to TD in *C* vs. to top of BU2, (followed by 2-layer readout network), in *D*]. The model in *D* must extract all outputs simultaneously.

A pivotal requirement in learning to extract scene structures is the ability to deal with novel configurations of components, properties and relations (25). This ability, termed “combinatorial generalization,” plays an important role in scene understanding, as well as other cognitive tasks (4). Combinatorial generalization arises even in simple configurations like the example in Fig. 3*A*, illustrating combinatorial generalization in our so-called Persons task. Each image in this dataset contained two “persons,” with a number of properties (hairstyle, glasses-type, shirt, etc.), and the task was to recover the identity of the two persons and their associated properties. We tested combinatorial generalization by excluding a number of person–property pairs from the training set (e.g., Person-7, glasses-type-3 were never presented together to the model). Model evaluation was then divided into a noncombinatorial test, using random new feature configurations, but without using any of the excluded pairs, and a combinatorial generalization test, where each test image contained at least one excluded person–property pair. Severe limitations of combinatorial generalization to such new combinations were demonstrated by network models in both visual and nonvisual tasks (26–28). We compared the generalization of the BU-TD model with a nearly identical model, which did not use task selection. The difference between the models is shown schematically in Fig. 3 *C* and *D*.

Fig. 3*C* shows the BU1-TD-BU2 unfolded backbone, with arrows pointing to the location where the instruction was provided. To make the comparison between models as close as possible, the task-unguided model (in D) was designed to be identical to the BU-TD model, except for the instruction vectors that were provided only at the last layer of the BU part. Model (C) is guided by the instruction, while (D) must extract simultaneously all the properties of the two persons in a single pass, and then select the instructed output, using a 2-layer readout net (*Materials and Methods, Readout Selection in Combinatorial Generalization*). The training of the two models is closely similar, since, if the TD instructions given to the two models are the same, the correct outputs are identical.

The comparison used two training set sizes: a “sufficient” set (1,600 images in the Persons task), the smallest set for which the BU-TD achieved accuracy criterion (85% on the excluded set); and “extended” set, defined as four times larger. This allowed us to compare both final performance as well as the amount of training required to obtain the final performance. Training terminated for each model according to a pre-set convergence criterion (improvement of <1% over 300 epochs). In this and all experiments below, we performed extensive hyperparameters search for all models and datasets, to find the best performing models within the search space of the parameters (*Materials and Methods, Hyperparameters Search*). The identical search over hyperparameters was used for all the models participating in the comparisons. Each experiment was repeated five times, over different randomly selected datasets and excluding different person–property pairs each time. Further training details in *Materials and Methods, Combinatorial Generalization*.

**Fig. 4.**
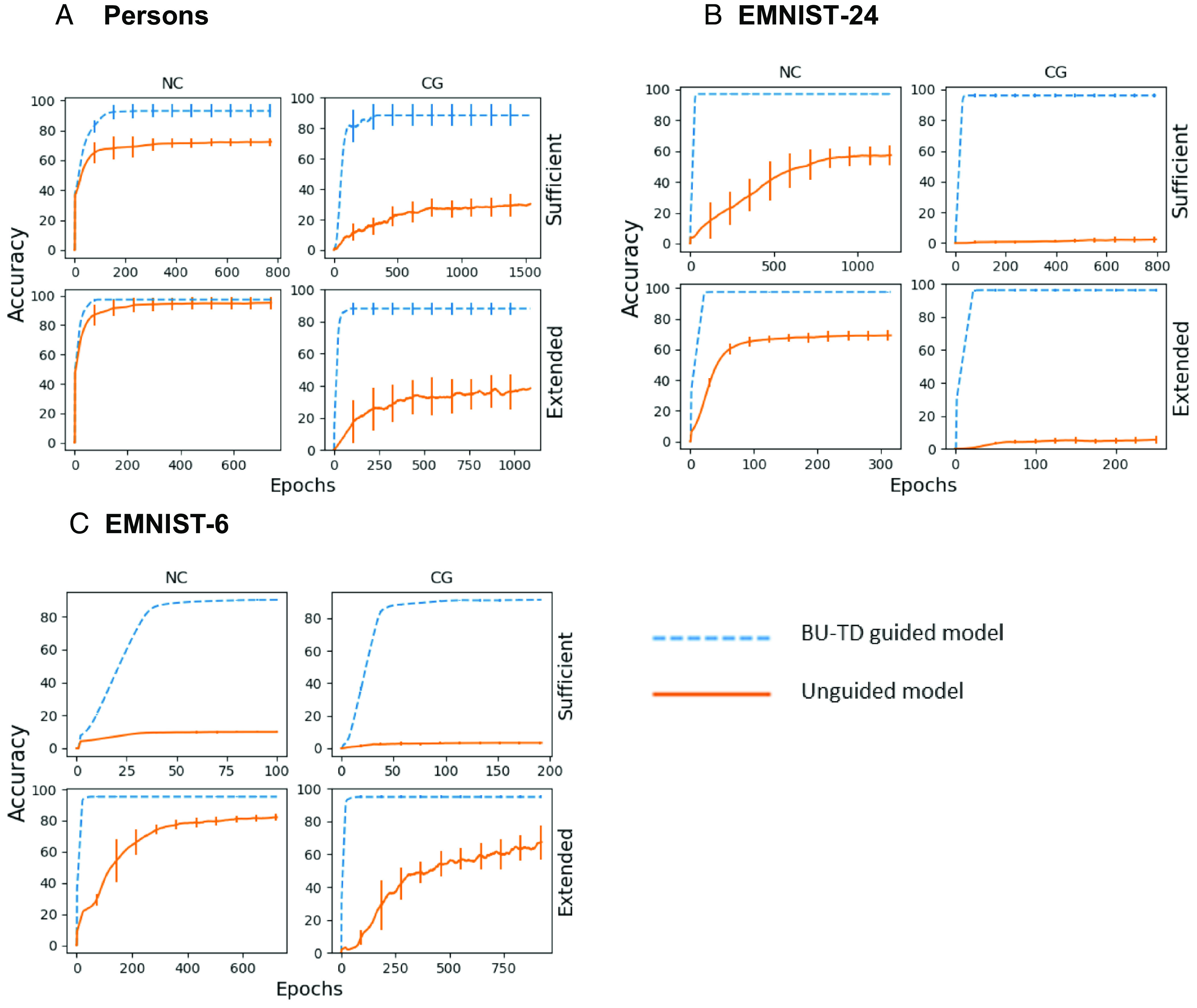
Combinatorial generalization results. (*A*) Persons task. *x* axis: training epochs, *y* axis: accuracy. Top row: sufficient data, bottom: extended data; NC: Noncombinatorial, CG: combinatorial generalization. Blue, dashed: guided BU-TD model, orange, solid: unguided. Each curve shows mean accuracy (averaged over 30 epochs) and SD (vertical bars, some too short to be visible) for the five repetitions. (*B*) EMINST-24 task, same notations as in *A*. (*C*) EMNIST-6 task, same notations as in *A*. For the unguided model, combinatorial generalization fails in *A* and *B*, and partially succeeds in *C*. For the guided model, combinatorial generalization is successful in all cases.

Results of the Persons test are shown in Fig. 4*A*. Throughout Fig. 4, noncombinatorial results (NC) were measured on the test images, combinatorial generalization results (CG) were measured on the novel person–property combinations only. The BU-TD model reaches high accuracy with the sufficient dataset and accuracy remains similarly high for combinatorial generalization. The unguided model eventually reaches high accuracy on the noncombinatorial test (extended set, NC); however, the accuracy in combinatorial generalization remains close to chance level (extended, CG).

A similar comparison was performed on the characters task (Fig. 4*B*). Images contained a set of 24 hand-written EMNIST characters (29), (selected from a total of 29). The task was to produce the right and left neighbor of each of the characters. The instructions used were <left-of, x> or <right-of, x> for a given character x in the image. One array of 24 characters was used as the excluded set: All successive pairs were excluded during training; further training details in *Materials and Methods, Combinatorial Generalization*. Similar to the Persons test, the unguided model showed slower learning, with little or no combinatorial generalization, while the guided model achieved high combinatorial generalization, with faster convergence (the sufficient dataset).

To further test the role of task guidance in generalization, we also tested a guided EMNIST model where the instruction was given in a BU instead of TD manner (as an additional channel together with the input). The model with BU instruction is still a guided model, and the comparison allows us to distinguish what makes the main contribution to combinatorial generalization: using guidance in general or using the guidance in a TD manner. The model reached comparable combinatorial generalization (but required substantially more training examples), details in *Materials and Methods, Combinatorial Generalization*, supporting the role of guidance in combinatorial generalization, regardless of model details.

We finally tested whether combinatorial generalization may emerge in the unguided model by sufficiently simplifying the task. As shown in Fig. 4*C*, generalization became partially possible when the EMNIST task was reduced to six characters (CG, extended training), but it was still lower than the BU-TD model, and learning was significantly slower. Increasing the fraction of excluded pairs caused accuracy of the unguided model to drop sharply (3% at 60% exclusion), but the guided model remained high, for both EMNIST-6 and EMNIST-24, details in *Materials and Methods, Combinatorial Generalization*. Repeating the EMNIST-6 tests of the unguided model, but extracting all the outputs simultaneously (without readout selection) produced the same results as the selective readout model (*SI Appendix*, *Simultaneous Outputs Model*). The combinatorial generalization comparisons show an inherent generalization advantage of the BU-TD sequential processing guided by early selection, compared with late selection (at the end of BU2) or simultaneous processing without selection. Additional comparisons of the current model with alternative models in *SI Appendix*, *Comparisons with Alternative Models*).

#### Compound instructions.

In extracting object properties in the experiments above, properties were extracted one property at time. We found that training to extract individual properties of an object can generalize spontaneously to the extraction of more than a single property at a time, without additional training (30). For example, combining the instructions for extracting glasses-type and hair-style (Fig. 3*A*) into a single instruction vector would lead to the correct extraction of both properties of the selected person. Compound instructions can be also extended to a larger number of properties with high accuracy and without hampering combinatorial generalization. Using such compound instructions reduces the number of extraction cycles and accelerates the extraction of scene structures for both full and guided structures.

## Discussion

The results show that the BU-TD model can extract scene structures of interest to the viewer, which, like in human perception, evolve sequentially over time, in a goal-directed manner. The model is consistent with the two streams of the cortical structure, and, combined with a mechanism for selecting the next TD instruction, it extracts structures of interest, with broad generalization to new configurations.

The results and approach can be contrasted with the main current alternatives. The closest approach to our scene interpretation model is the extraction of scene graphs (31). However, unlike scene graph models, our model is guided to extract selected scene structures. The specified structures can also include quantifiers, which are not used in scene graph models. Another possible alternative is based on the recent development of large vision-language (VL) models (32, 33). For example, VL question-answering (VQA) models offer the possibility of using natural language queries to guide a visual model toward scene structures of interest. However, compared with our model, current VQA and VL models in general have severe limitations in extracting scene structures (34–36) as well as in the use of quantifiers (24, 37), which limit their use for scene interpretation.

**Fig. 5.**
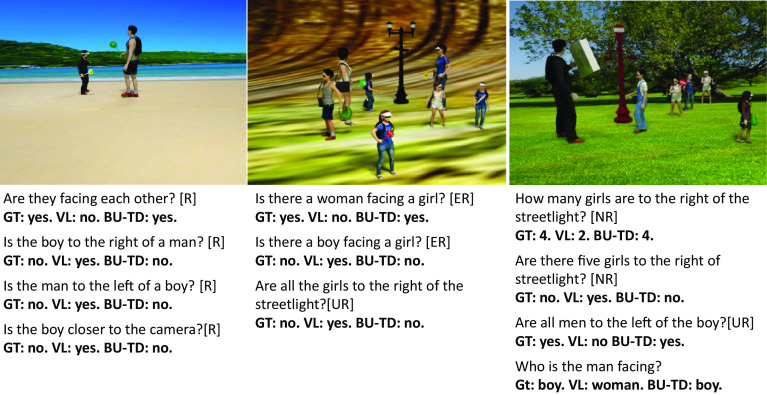
Visual examples of structures and tests. Examples of tests listed in Table 1, and the text used in the vision-language (VL) model to test for the presence of the structures in the image. In all shown examples, responses of the VL model were incorrect. GT is the correct answer (ground truth). Further details in *SI Appendix, Comparisons with Alternatives Models*.

For example, tests of VL models on their ability to understand different types of relationships, attributes, and order information (34) found that the tested models failed to perform beyond chance level on tasks requiring compositional understanding. Similar tests have also shown that current VL models have difficulties with the use of quantifiers. For example, studies in ref. 37 showed that current VQA models have difficulties with number quantifiers, especially in counting tasks involving objects with their properties (e.g., “how many boys are wearing a hat?”), which our model handles well (e.g., *SI Appendix*, Fig. S4). The VL models that failed in scene interpretation often performed well in various vision-language tasks, but the limitations were revealed by datasets and tests focusing on the recovery of scene structure (34–36). Simple examples of the limitations above are illustrated and compared with the BU-TD model in Table 1 and Fig. 5; further details in *SI Appendix*, *Comparisons with Alternative Models*.

**Table 1.** Average accuracy comparisons of the guided BU-TD model and a VL model (33)

	BU-TD (%)	VL fine-tuned (%)
Objects [O]	99.6	99
Relations [R]	94.4	59.9
Universal + Relation [UR]	91.2	53
Numeric + Relation [NR]	72.3	45.8
Existential + Relation [ER]	79.6	50.2

Prior to testing, the VL model was fine-tuned to recognize the rendered objects and persons in the dataset (Objects [O]). “Relations” [R] tests relation recognition in images containing a single pair of objects; [UR] tests a relation combined with a universal quantifier (e.g., “All”); [NR] combines a relation with numerical values (e.g., “How many”); [ER] tests a relation combined with an existential quantifier (e.g., “There is”). Examples are illustrated in Fig. 5.

Our findings regarding combinatorial generalization have potential implications to the role of attention processes in human vision. In theories of human perception, the main role of selective attention [though not the only one (38)] is related to capacity limitations (39, 40): visual scenes may contain more items than can be processed at any one time, and attention processes deal with this capacity constraint by selecting relevant and filtering out irrelevant information. Our findings demonstrate another major function for the use of early visual selection, in terms of affording combinatorial generalization. As shown, the roles are independent in the sense that even when capacity is sufficient to deal with all the relevant information, early selection offers a substantial advantage in terms of combinatorial generalization.

The extraction of scene structures by the model is guided by TD instructions that are initiated at the top-level parts of the network and can therefore be determined by a combination of visual and nonvisual information. This combination that takes place at each cycle of the interpretation process allows nonvisual, higher level representations to participate in the extraction of meaningful scene structures. The current model focuses on the visual part of the interpretation process and does not include a network model of the higher-level process of selecting the TD instruction (*SI Appendix*, Algorithm S2). In the model simulations the goals were given externally, but in human perception they originate internally and evolve over time during scene perception (2, 9, 12–14). Their dynamic generation should eventually be a part of the overall scene perception model. In the future, it will be of interest to extend the model to a unified network model, which combines the perceptual part with the more cognitive aspects involved in selecting the guidance, as a step toward a model for integrating visual and cognitive processes in scene analysis.

## Materials and Methods

### Scenes Dataset.

The dataset consists of computer-generated scenes with human figures and objects, created using Makehuman graphics (http://www.makehumancommunity.org/), an open-source tool for character making. The people come from four classes (woman, man, girl, boy), can have different poses, and different properties. We included eight different outfits, optional sunglasses, optional hat, four hairstyles, possibly carrying an object (backpack, shoulder bag), or holding an object (bag, hammer, tennis racket, signal, sword). Scene objects of different types, sizes, and colors can be placed in the scenery (bench, chair, trashcan, streetlight, and tree).

Relations between persons used in the main experiments included spatial relations (“left,” “right,” “in front,” “behind”), “facing,” “touching,” “closest” (to another person or an object) and “obstructing” (blocking the path of another person, within a given distance). A person can interact with an object via “holding” and obstructing relations, and relations between objects include the spatial relations and the “on” relation. Images were generated by sampling a scene graph, i.e., sampling the objects and their properties, locations in the scene, and relations between them. The images were rendered using Blender (https://www.blender.org/), with given camera and lights locations. The generated images have full annotations of all components, properties and relations, and have complete segmentation maps. These annotations were used in training all the TD instructions used in the extraction process. Below are training details and accuracy obtained in training and test, measured by segmentation IoU (intersection-over-union), and by average classification accuracy (of components, properties and relations).

Number of images used: Train: 12,000, Test: 3,000.

*Expansion network:* Number of examples (an “example” is a result of a TD instruction applied to an image): Train: 608,480, Test: 153,371.

Results (train/validation): IoU at TD: 0.617/0.604, accuracy at BU2: 0.846/0.831

*Expansion network with persons’ priority:* Number of examples: Train: 690,450, Test: 173,977

Results (train/validation): IoU at TD: 0.612/0.6, accuracy at BU2: 0.852/0.838

*Elaboration network:* Number of examples: Train: 2,121,340, Test: 535,799

Results (train/validation): IoU at TD: 0.938/0.937, accuracy at BU2: 0.933/0.928

### Guided Structure Extraction and Selecting the Next TD Instruction.

The procedure for selecting the next TD instruction is a recursive process that handles one node at a time (starting at a root node if a root node exists, and arbitrary node otherwise), building upon the algorithm in ref. 23. The persons and scene objects (e.g., a bench or a streetlight) are extracted one by one using the <extract-next> instruction. The algorithm proceeds along the nodes in the target structure, and it guides the model to extract people and objects with their properties and relations that will correspond to the structure described by the target graph. If the class of the extracted person or object corresponds to the required class of the current node (e.g., girl), the process checks the properties and relations’ requirements of the node. The relevant properties are extracted and validated by providing the appropriate TD property instructions to the elaboration network, and the relevant relations are extracted and validated using the expansion network, which retrieves the persons and objects required by the relations in the graph (e.g., facing). Spatial relation instructions (e.g., right-of, behind) extract people and scene objects, and relations such as holding or on are used to extract tools and objects held by people or placed on other objects. Once all the node’s tests are completed, the process repeats for the next node, determined by a depth-first traversal. The retrieved information is saved and updated in an array, where each entry represents data of one component (object or person). The retrieved components that fulfill the requirements are paired with the corresponding components in the target graph, so that subsequent tests will be applied to the correct objects (*SI Appendix*, Fig. S4*B*). The number of required objects is set according to quantifiers (e.g., “all,” “three”), or by the need to evaluate a property that depends on the entire object set (e.g., how many). After the node and the following nodes in the recursion are tested and validated in the image, tests are applied, if needed, to verify the so-called node’s set requirements (e.g., counting the objects in the set and making quantity comparisons). The recursive process is terminated either when the full target graph is grounded in the image as required, or when no alternatives are left to check, which may occur already after a partial evaluation of the image, e.g., no objects of a required class were detected.

Pseudocode description of the algorithm for selecting the next TD instruction and extracting a structure of interest is given in *SI Appendix*, Algorithm S2.

### Combinatorial Generalization.

The BU-TD network for the Persons experiment (Fig. 3*A*) was based on Resent-18, and for the EMNIST experiments, which is a simpler task, on Resnet-6. In the Persons dataset, the Sufficient dataset had 1,600 training images from which we generated 16,000 training examples (person–property pairs), with additional 1,180 noncombinatorial test examples and 1,178 combinatorial test examples. The Extended dataset was four times larger, with 6,400 training images and 63,940 examples, with additional 1,174 noncombinatorial examples and 1,178 combinatorial test examples.

The EMNIST-24 sufficient dataset had 10,000 images, using 16 examples (character-direction pairs) per image, resulting in 160,000 training examples, and additional 2,000 noncombinatorial and 2,000 combinatorial generalization test examples. The Extended dataset was again four times larger in terms of images and training examples. We excluded testing the “right-neighbor” TD instruction at row-end, which has no neighbor, similarly for left-neighbor we excluded the first character in a row.

The EMNIST-6 sufficient dataset used 2,500 images, five examples per image, total 12,500 training examples. The extended dataset had 10,000 training images, 50,000 training examples. Both sets had additional 2,000 noncombinatorial and 2,000 combinatorial test examples.

We used the same losses on all models: the first (top of BU1) was an occurrence cross-entropy loss (i.e., which characters or persons appeared in the image), the second (top of BU2) was the task loss (a person’s property detail, or character right/left-of neighbor).

Bottom–up guidance: We repeated the tests in another model (single repetition), similar to the guided BU-TD model, except that the instruction was provided in a BU manner, together with the input image, as an additional input channel. This was accomplished by learning an embedding of the instruction into a vector of the same size of the image.

### Readout Selection in Combinatorial Generalization.

The readout in the unguided model uses the top layer of BU2 and the embedded instruction representation as input, one fully connected intermediate layer (Relu connections), and a final linear readout layer.

### Hyperparameter Search.

We performed extensive hyperparameter search for all combinatorial generalization models. For the EMNNIST character experiments (Fig. 3*B*), we used 30 parameter combinations. The parameter set was a combination of an optimizer (SGD, stochastic gradient descent with momentum and ADAM), learning rate (six values: 0.0001, 0.001, 0.002, 0.05, 0.1, 0.2), batch size (10, 32, 48), and weight decay (0.0001, 0.0002). Similarly, for the Persons experiments 18 combinations were used (fewer batch sizes). The search was used to find the best performing models within the search space of the parameters, and the identical search over hyperparameters was used for all the guided and unguided models participating in the comparisons. The search repeated five times for the five different folds.

## Supplementary Material

Appendix 01 (PDF)

## Data Availability

Code and images data have been deposited in Github (https://github.com/liavassif/BU-TD) (41).
