# Supplementary material for: Human-like scene interpretation by a guided counterstream processing
Source: Proc Natl Acad Sci U S A. 2023 Sep 28;120(40):e2211179120. doi: 10.1073/pnas.2211179120 (PMC10556630; doi:10.1073/pnas.2211179120)
Supplement: Supplementary file 1 — Appendix 01 (PDF) [file pnas.2211179120.sapp.pdf]

Supplementary Information for

Human-like scene interpretation by a guided counter-streams processing

Authors: Shimon Ullman, Liav Assif, Alona Strugatski, Ben-Zion Vatashsky, Hila Levi, Aviv Netanyahu, Adam Yaari

Corresponding author name: Shimon Ullman,
Email: Shimon.Ullman@weizmann.ac.il

This PDF file includes:

Supplementary text
Figures S1 to S4
Algorithms S1 to S2
Supplementary references

Supplementary Information Text

Cross-streams lateral connections and losses

There are two sets of cross-stream lateral connections, in the BU→TD and TD→BU directions. We compared two alternative schemes, one using additive, and the other multiplicative connections. Consider a unit $\bar{x}_{i,k}$, in channel *i* and location *k* of a given layer on the TD stream (upper-bar denotes variables on the TD stream). Its total additive input *I* equals $\bar{I}_S + I_L$, where $\bar{I}_S$ is the input along the TD stream, and I_L the lateral contribution from the BU stream. One form for I_L we used was convolutional, using: $I_{L_k} = \Sigma_c\, w_{i,c} x_{c,k}$, where the lateral input to $\bar{x}_{i,k}$ comes from all units $x_{c,k}$ at the corresponding location in the corresponding layer on the BU stream, across all channels. The weights are independent of the location *k*, but depend on the target channel *i*. In a simplified case (used in the expansion and elaboration networks), the inputs to a target channel *i* on the TD stream came from the single channel *i* on the BU stream. In the multiplicative scheme $I_L = \Sigma_c\, w_{i,c} x_{c,k}$ is used in a multiplicative form: $\bar{x}_{i,k} = \bar{I}_S * I_L = \bar{I}_S * \Sigma_c\, w_{i,c} x_{c,k}$. This form can also be combined with an additive term, $\bar{x}_{i,k} = \bar{I}_S * I_L + \alpha I_L$ (with α a learned parameter). Comparisons on a number of tasks suggest that for the TD→BU direction, a combination of additive and multiplicative was better than either scheme on its own, for the BU→TD direction the additive scheme was sufficient. The TD→BU cross-stream connections change the response properties of units along the BU stream and modify its processing, depending on the TD instructions.

The main loss used in training was cross-entropy at the end of BU2. Additional losses can be naturally used at the top of BU1 and at the end of the TD stream. For instance, in learning the instruction <right-of, z> in an array of EMNIST characters (Fig. 3B, main text), the loss at BU1 was cross-entropy classification loss of all the characters present in the image, and the TD loss was a segmentation L2 loss of the reference letter z.

Extracting full scene structure

An example of extracting full scene structure is shown in Fig. S3. The process starts by extracting all scene components (persons and scene objects) by invoking the expansion network (with 'extract-next' instruction), until 'no component' is returned, and storing them in an array. The second step is querying the properties for all components using the elaboration network and updating the results for the corresponding component in the array. The particular set of property instructions is determined according to the item type (e.g. the 'clothes' instruction is activated only for persons). The final step is getting the relations between the components (for the spatial relation we use 'right' and 'behind' (for immediate right and immediate behind) and omit their complementary 'front' and 'left'). For each component, the expansion network is activated multiple times, each time with a different relation instruction. The particular set of relations is determined according to the component type (e.g. 'facing' instruction is activated only for persons). An 'auxiliary-object' (e.g. hammer, hand-bag) is retrieved from the image only by an instruction of a corresponding relation ('holding' or 'on') and not by the 'extract-next' instruction. The retrieved 'auxiliary-object' is added to the array and its properties are extracted (using the elaboration network), and added. For other relations used by the expansion network (e.g. <Facing, person-1>), the target scene component should be one of the components already represented in the array. The component in the array with maximal intersection over union (IoU) overlap is selected. If the maximal IoU is below a threshold, the new component is added to the array.

A schematic 'pseudo-code' description of the full-structure algorithm is given in Algorithm S1. 'Flag' in the code refers to TD-instruction. The functions *get_item_by_flag* and *get_prop_by_flag* invoke the expansion network and elaboration network, respectively.

Providing goals to the model

The target structure used as a goal may be given in a non-explicit manner, e.g., it may be stated in natural language, in which case a parsing stage will be required to construct the structure of interest (1, 2). Similar to the present model, the method in (1) applies a sequential program to the image, but only after extracting a full scene analysis, and without producing a target structure. The instruction selection algorithm used by the model goes beyond graph structures, and deals also with logical connectives and quantifiers (Fig. S4A). A scene can also be interpreted without a specific initial goal, in which case our partial solution is to use guidance by general priorities, to increase the probability of extracting as early as possible information likely to be of interest. In particular, we use instructions to extract early in the process the presence of people, their interactions, and interactions between people and objects (Fig. S4C). In addition to TD priorities, bottom-up saliency could also affect the order of information extraction from the image (3), and the two can be combined by BU-TD integration (4)

Simultaneous Outputs model

The simultaneous model used a standard matrix representation for the output, with the task 'right-of', where activating output unit *M(i,j)* means that character *j* is the right-neighbor of character *i*. In this representation, the output units representing pairs excluded from training were never activated during training, and therefore activation of the correct output units cannot be expected. We tested whether the correct answers were in fact encoded in the top layer of the network prior to the activation of the *M(i,j)* units, by training a 2-layer readout from this last layer, to predict the correct output. The readout training took place only after the full training of the main network was completed. Using the readout network, we found that the parallel-learning version reached the same generalization accuracy as the sequential selective readout. The sequential selective readout and simultaneous outputs networks are equivalent in accuracy, but the selective readout has the advantage that results for the pairs excluded during training can be read out in a manner that is not obtained by the more standard matrix representation or in a multi-branch architecture.

Comparisons with alternative models

As a direct comparison with our model, we tested whether VL models can extract scene structures when applied to our data set of generated scenes. For this purpose, we compared our model and a large vision-language model on the same images, taken from our scenes data set. We tested the ability to extract relations and to deal with quantifiers.

Extracting scene structures

Since our data set contained rendered persons and objects, which may not be familiar to existing VL models, we first trained a vision-language model to recognize the persons and objects in our scenes data set. Training the model was obtained by fine-tuning the BLIP model (the LM architecture for VQA tasks, BLIP w/ ViT-B and CapFilt-L) (5) combined with LoRA (6), which we used to avoid forgetting effects in the original model. Training for object recognition used a total of 4,500 training images, each containing a single rendered object out of 9 (boy, girl, man, woman, bench, chair, trash can, streetlight, tree). We trained the model for 15 epochs reaching classification accuracy of 99%. Testing the validation set of VQA2 resulted in similar accuracy as the original model, 83.52%, showing that there was no forgetting in the original model.

We then proceeded to test the model on extracting relations between scene components in images containing two objects ([R] in the main Fig. 5). Relations were tested using the standard procedure similar to so-called VL-CheckList (7). During testing, images were combined with questions such as: 'is the man to the left of a boy?', 'is the woman closer to the camera?' or 'are they facing each other?' etc.

Testing relations and quantifiers

We tested number quantifiers, combining scene images with questions such as 'how many women are to the left of a bench?'. The universal 'All' quantifier was tested along with relations by combining scene images with questions such as: 'are all the men to the right of the bench?', which could be either correct or false depending on the image. Existential quantifiers were tested on images with several persons using questions such as 'is there a girl facing a boy?'. Unlike simple relations, this test requires searching and verifying the selected structure. The number of test images were: 300 for number quantifiers, 600 for the 'Facing' relation, and 1,000 for each of the other relations, universal, and existential quantifiers.

Comparing models on combinatorial generalization

We also compared our model with a vision-language model on their performance on the EMNIST-6 combinatorial generalization task, described in the main paper, section 'Combinatorial generalization'.

We used the BLIP model (BLIP w/ ViT-B and CapFilt-L) fined-tuned for VQA tasks (5)

The model was trained to perform the tasks of identifying the left-neighbor or right-neighbor of EMNIST characters. Training was done on a dataset of 50,000 images with 6 characters in each image, and testing on additional 2,000 images. The right-of task was trained on half of the characters, and the left-of on the other half. During testing, non-combinatorial tests used the same characters that the model was trained on, while the combinatorial generalization test used characters not seen during training, e.g., the instruction (left-of, 6), which was a new combination, not used in training. The guided model was trained to extract a single relation at a time, i.e., the left or right neighbor of a given character in the image. The unguided version was trained to recover all relations simultaneously, that is, the neighbors of all characters in the image.

Results:

The guided model achieved an accuracy of 91% for non-combinatorial testing, and 90% for the combinatorial generalization test.

The unguided model achieved an accuracy of 98% for non-combinatorial testing, and 0.3% accuracy for the combinatorial generalization test. (Chance level is about 3%). The results show that the combinatorial generalization superiority of a guided model compared with an unguided model, is not specific to our model, but is shown by state-of-the-art VL model applied to our data set.

Transformer model
We also tested combinatorial generalization using a visual transformer model (without a language part), instead of the convolutional neutral network used by our BU-TD model. The ability of large models to extract compositional structures sometimes decreases with the size of the models (8), we therefore used models of two sizes, to evaluate the effect of model size on the combinatorial generalization performance. We used the CCT transformer (9). The smaller transformer used 2 convolutional layers followed by 2 transformers, embedding size was 128. This basic component was repeated 3 times, similar to the BU1, TD, and BU2 components used in the original non-transformer model. The larger model had 7 transformer layers instead of 2 and embedding size of 256. For the guided model, the task and argument instructions were given using additional input tokens, provided to the model as additional inputs. The unguided model used a similar structure but without the task and argument tokens.

Results:
The guided transformer model achieved high accuracy for both the non-combinatorial and combinatorial generalization (80%).
The unguided transformer model resulted in a similar non combinatorial accuracy, but low combinatorial generalization (40%).
We further compared the results with the larger model, with more transformer layers and a larger embedding size. The guided model achieved high accuracy results (84%), while the unguided model obtained high accuracy on the non-combinatorial test, similar to the smaller model, but low combinatorial generalization accuracy (23%). Results of the transformer models were similar to the results reported in the main paper with non-transformer models, and also showed that for these models, increasing model size does not necessarily increase combinatorial generalization accuracy.

The models will be made publicly available.

Figures

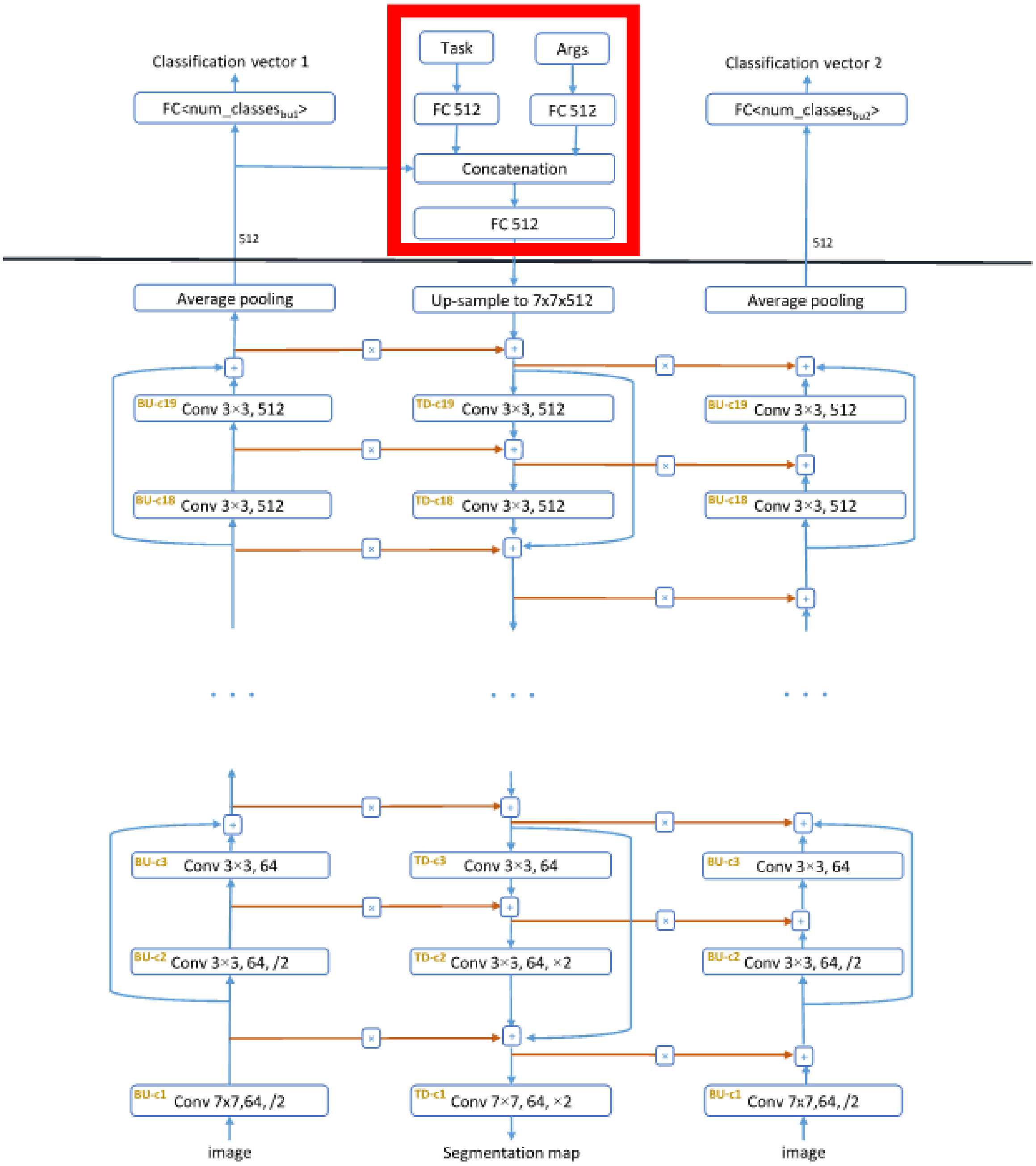

Fig. S1. Full structure of a BU-TD network. The BU network in this example is a ResNet-18. The red frame surrounds the part where the TD instruction is provided. The model is shown here in its unfolded form used for training (Fig. 1D), with its BU1, TD, BU2 components. Each layer shows the layer identifier (such as BU-c3), the operation it performs (Conv for convolution, FC for fully-connected) and dimensions; small boxes with + or x indicate additive or multiplicative operation. The cross-stream connections are described in 'Cross-streams lateral connections and losses' above.

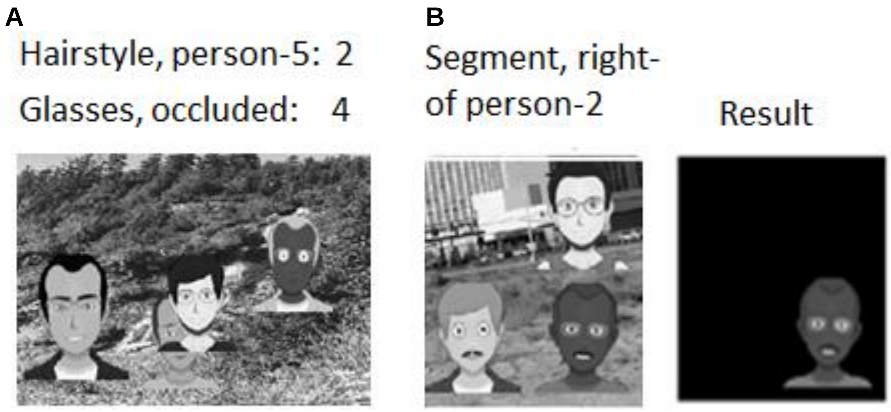

Fig. S2. Providing TD arguments. (**A**) Argument specified by an object (‘person-5’) or property (‘Occluded’), i.e., classify the glasses of the occluded person. The selected person or property were represented by 1-hot vector from a list of possible arguments. The tasks in the example were 'hairstyle' and 'glasses', results were 'hairstyle 2' and 'glasses type 4, which are correct. (**B**) Argument specified by a relation (‘Right-of); segmentation (shown in ‘Result’) is produced at the end of the TD stream.

Location argument (not shown in figure) is specified by a center location or by an image region in a coarse TD map; it can also be specified in a BU manner by a spatial map supplied together with the input image. TD instructions and output results are shown above the images.

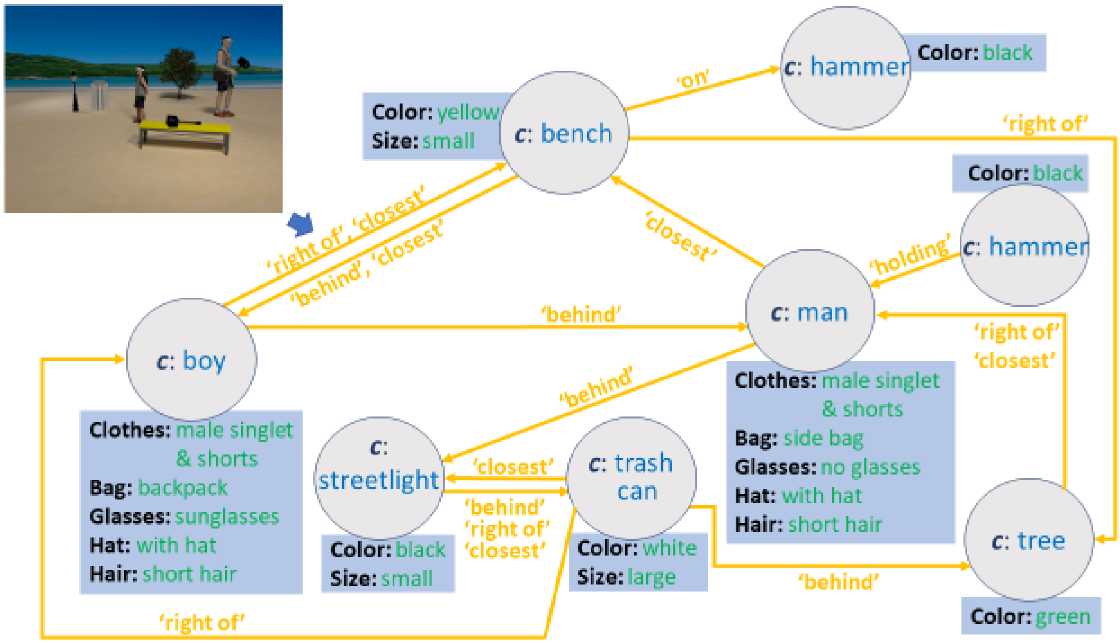

Fig. S3. Extracting full scene structure. The graph illustrates all the components, properties and relations extracted by the sequence of TD instructions produced automatically by the full-structure algorithm (Algorithm S2 below). Prediction accuracy of the objects, properties and relations is similar to the results in (Materials and methods, 'Scene data set') for small graphs, and reduces with the graph size.

A

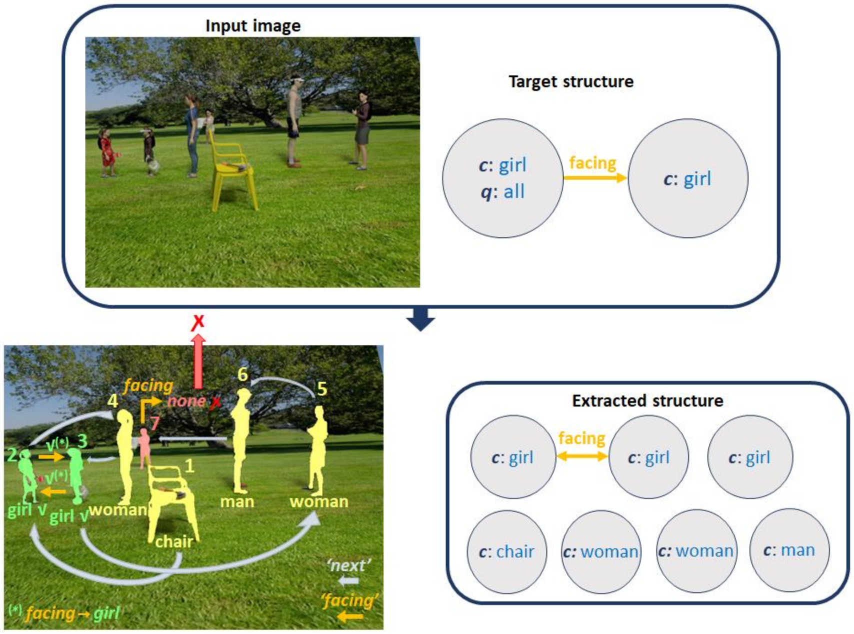

Quantifier: 'All'

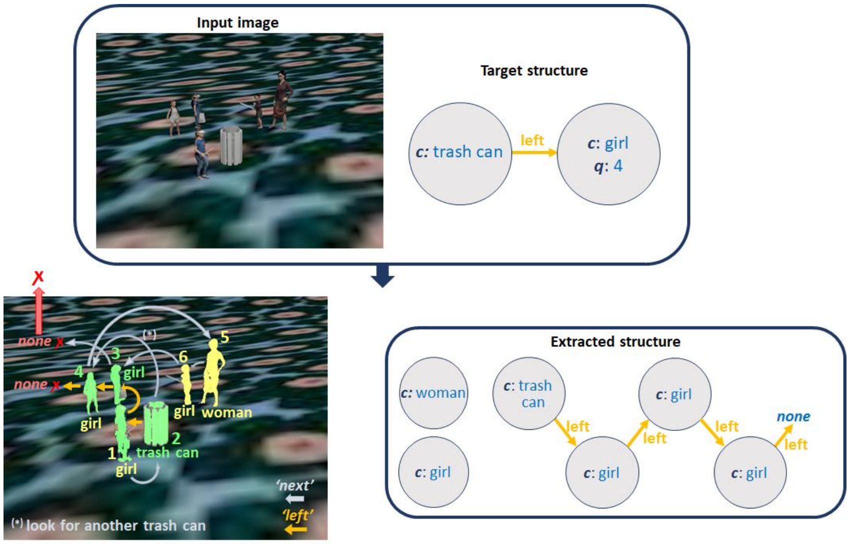

Quantifier: '4'

B

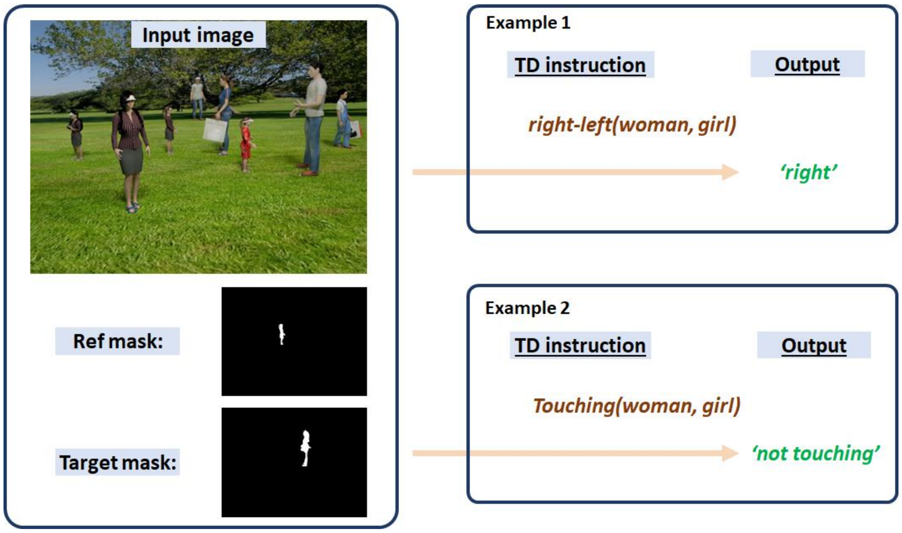

C

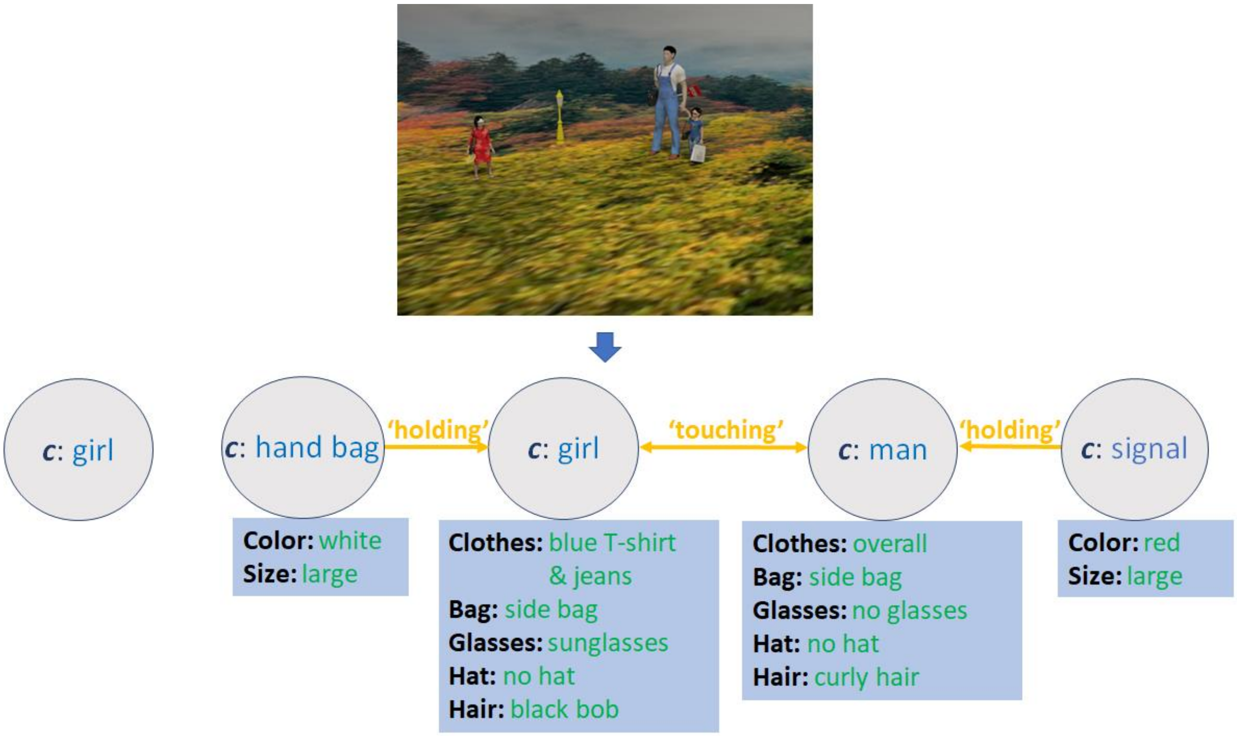

Fig. S4. Guided extraction: quantifiers, grounding, default priorities. (A) Goals containing quantifiers and number. Each example shows the input image and target structure (top part), the automatically generated extraction process (bottom left), and the extracted structure (bottom right). Top example: the goal is to find all girls that are facing girls. The girls marked in green are facing girls, the girl in red is not. Bottom example: testing if there are 4 girls to the left of the trash can.

The process identifies 3 girls to the left of the trash can, it then searches for an alternative trash can, and fails.

Grey arrow: expansion using ‘extract-next’; orange arrow: expansion using a relation; numbers indicate the order of components extraction (by both next-component and relation); √, or green components: consistent with the requirements of the target structure. Yellow: irrelevant components, χ or rose color component: violates a requirement; (*) a comment (marked at the image-bottom). In the top figure, (*) marks the location of successful girl-facing-girl instructions. In the bottom example, (*) marks the last TD instruction, which is to find another trash can. **(B)** Grounding: computing relations using the grounding established between extracted components and their image. The relations right-left(x, y) and touching(x, y) are applied to the girl and woman (holding a bag, in the scene center), extracted earlier in the process (Fig. 2, main text). **(C)** Default priorities. A goal is not provided, and the extraction process uses default priorities for interacting people. People in the scene are extracted before scene objects using <next-person> TD instruction. When interacting people are found, the TD instructions to the elaboration network are used to extract their properties and held objects; these properties are not extracted for non-interacting people. Held-objects by non-interacting people are the next priority (not shown in figure).

Algorithm S1. Image full structure extraction

Given below is a pseudo-code description of the algorithm used to extract the full scene structure (all components, properties and relations).

```
Input: image
Result: image graph
Initialization: image_graph = [], item_class = 0, items_count = 0, scene_mask =zeros;
begin
    while item_class is ¬None ∧ items_count < MAX_ITEMS do
        [item_class, pred_mask] = get_item_by_flag(image, scene_mask, flag = 'next_item')^1;
        items_count = items_count + 1;
        if  item_class is ¬None then
            scene_mask = scene_mask ∨ pred_mask;
            overlap_ind = get_item_ind_by_overlap(pred_mask, image_graph);
            if overlap_ind is None then
                item['class'] = item_class;
                item['mask'] = pred_mask;
                item['child_nodes'] = [], item['child_rels'] = [];
                item['parent_nodes'] = [], item['parent_rels'] = [];
                for prop_type in prop_list^2 do
                    item[prop_type] = get_prop_by_flag(image, pred_mask, flag = prop_type)^3;
                end
                image_graph.append(item);
            end
        end
    end
    for item_ind in length(image_graph) do
        for rel in rels_list^4 do
            [rel_item_class, rel_pred_mask] = get_item_by_flag(image, image_graph[item_ind]['mask'], flag =
        rel)1;
            child_ind = get_item_ind_by_overlap(rel_pred_mask, image_graph);
            if rel in tool_rels ∨ child_ind is None then
                new_item['class'] = rel_item_class;
                new_item['mask'] = rel_pred_mask;
                new_item['parent_nodes'] = [], new_item['parent_rels'] = [];
                for prop_type in prop_list^2 do
                    new_item[prop_type] = get_prop_by_flag(image, rel_pred_mask, flag = prop_type)^3;
                End
                child_ind = length(image_graph);
                image_graph.append(new_item);
            End
            image_graph[child_ind]['parent_nodes'].append(item_ind);
            image_graph[child_ind]['parent_rels'].append(rel);
            image_graph[parent_ind]['child_nodes'].append(child_ind);
            image_graph[parent_ind]['child_rels'].append(rel);
        end
    End
End
```

[1] *get_item_by_flag* function invokes the expansion network
[2] property list is adopted according to item type (e.g. 'clothes' is relevant only for persons)
[3] *get_prop_by_flag* function invokes the elaboration network
[4] relation list is adopted according to item type (e.g. 'facing' is relevant only for persons)

Algorithm S2. Guided scene structure extraction

Given below is a pseudo-code description of the algorithm for the guided extraction of a specified scene structure.

```
Input: query_graph, image
Result: query answer, retrieved structure graph
Initialization: workMem['current_node'] = first_parent_node;
Run [success, answer] = getGraphAnswer :
 Begin
  | current_node = workMem['current_node'];
  | Node parameters: p: properties, r: relations, f: property type, g: property of a set;
  | last_item, no_items, no_child_items =False; mask =zeros;
  |  while ¬no_items ∧ ¬last_item do
  |  | if child_node then
  |  |  | item = workMem['item'];
  |  |  | last_item = True;
  |  | else if is_super_node[5] then
  |  |  | [item, last_item] = get_item_from_saved_sub_nodes;
  |  | else if exist(saved_detected_items) then
  |  |  | [item, last_item] = get_item_from_saved_detected;
  |  | Else
  |  |  | [item, no_items] = get_item_by_flag(image, mask, flag = 'next_item')[6];
  |  |  | mask = mask ∨ item['seg'];
  |  | End
  |  | if ¬empty(p) then
  |  |  | for p in p do
  |  |  |  | p̂ = get_prop_by_flag(image, item['seg'], flag = f_p)[7];
  |  |  |  | success = p̂ == p;
  |  |  |  | if ¬success then break end
  |  |  | End
  |  |  | if ¬success then continue end
  |  |  End
```

[5] A 'super node' includes other nodes ('sub nodes')

[6] *get_item_by_flag* function invokes the expansion network

[7] *get_prop_by_flag* function invokes the elaboration network

```
        if ¬empty(f) then answer = get_prop_by_flag(image, item['seg'], flag = f)Error! Bookmark not defined. end
        if empty(r) then
            if exist(next_parent_node)
                workMem['current_node'] = next_parent_node;
                Run [success, answer] = getGraphAnswer;
            End
        Else
            for r in r do
                child_mask = item['seg'];
                while ¬no_child_items do
                    if empty(item[r_item]) then
                        [item[r_item], no_child_items, answer] = get_item_by_flag(image,  child_mask, flag = r)Error! Bookmark n
                        ot defined.;
                    End
                    child_mask = item[r_item]['seg'];
                    success = ¬no_child_items;
                    if success then
                        workMem['current_node'] = next_node[8];
                        workMem['item'] = child_item;
                        Run [success, answer] = getGraphAnswer;
                        if success ∧ (#success_child_items == #required_child_items[9]) then break else success == False end
                    End
                End
                if ¬success then break end
            End
        End
        if success ∧ (#success_items == #required_items[9]) then break end
    End
    if success ∧ ¬empty(g) then answer = g(valid_items) end
    if success ∧ comp_num_en ∧ is_checked(comp_node) then answer = comp_num[10](valid_items, comp_items, comp_type); end
    if success ∧ is_sub_node then save_for_super_node(success_items); end
    return [success, answer]
End
```

[8] Either child node or next unvisited root node of a subgraph
[9] According to quantifiers and other requirements
[10] Compare number of valid items between nodes ('same', 'fewer', 'more')

SI References

1. K. Yi, *et al.*, Neural-symbolic VQA: Disentangling reasoning from vision and language understanding. *Adv Neural Inf Process Syst* **2018-Decem**, 1031–1042 (2018).
2. B. Z. Vatashsky, S. Ullman, VQA with no questions-answers training in *Proceedings of the IEEE Computer Society Conference on Computer Vision and Pattern Recognition*, (2020) https:/doi.org/10.1109/CVPR42600.2020.01039.
3. S. Ullman, C. Koch, Shifts in Selective Visual-Attention - Towards the Underlying Neural Circutry. *Hum Neurobiol* **4**, 219–277 (1985).
4. J. H. Reynolds, D. J. Heeger, The Normalization Model of Attention. *Neuron* **61**, 168–185 (2009).
5. J. Li, D. Li, C. Xiong, S. Hoi, BLIP: Bootstrapping Language-Image Pre-training for Unified Vision-Language Understanding and Generation (2022).
6. E. Hu, *et al.*, LORA: LOW-RANK ADAPTATION OF LARGE LANGUAGE MODELS in *ICLR 2022 - 10th International Conference on Learning Representations*, (2022).
7. T. Zhao, *et al.*, VL-CheckList: Evaluating Pre-trained Vision-Language Models with Objects, Attributes and Relations (2022).
8. O. Press, *et al.*, Measuring and Narrowing the Compositionality Gap in Language Models (2022).
9. A. Hassani, *et al.*, Escaping the Big Data Paradigm with Compact Transformers (2021).
